# Increased Access to Care and Appropriateness of Treatment at Private Sector Drug Shops with Integrated Management of Malaria, Pneumonia and Diarrhoea: A Quasi-Experimental Study in Uganda

**DOI:** 10.1371/journal.pone.0115440

**Published:** 2014-12-26

**Authors:** Phyllis Awor, Henry Wamani, Thorkild Tylleskar, George Jagoe, Stefan Peterson

**Affiliations:** 1 Centre for International Health, Department of Global Public Health and Primary Health Care, University of Bergen, Bergen, Norway; 2 School of Public Health, College of Health Sciences, Makerere University, Kampala, Uganda; 3 Medicines for Malaria Venture, Geneva, Switzerland; 4 Global Health, Karolinska Institutet, Stockholm, Sweden; 5 International Maternal and Child Health Unit, Uppsala University, Uppsala, Sweden; University of the Stellenbosch, South Africa

## Abstract

**Introduction:**

Drug shops are a major source of care for children in low income countries but they provide sub-standard care. We assessed the feasibility and effect on quality of care of introducing diagnostics and pre-packaged paediatric-dosage drugs for malaria, pneumonia and diarrhoea at drug shops in Uganda.

**Methods:**

We adopted and implemented the integrated community case management (iCCM) intervention within registered drug shops. Attendants were trained to perform malaria rapid diagnostic tests (RDTs) in each fever case and count respiratory rate in each case of cough with fast/difficult breathing, before dispensing recommended treatment. Using a quasi-experimental design in one intervention and one non-intervention district, we conducted before and after exit interviews for drug seller practices and household surveys for treatment-seeking practices in May–June 2011 and May–June 2012. Survey adjusted generalized linear models and difference-in-difference analysis was used.

**Results:**

3759 (1604 before/2155 after) household interviews and 943 (163 before/780 after) exit interviews were conducted with caretakers of children under-5. At baseline, no child at a drug shop received any diagnostic testing before treatment in both districts. After the intervention, while no child in the non-intervention district received a diagnostic test, 87.7% (95% CI 79.0–96.4) of children with fever at the intervention district drug shops had a parasitological diagnosis of malaria, prior to treatment. The prevalence ratios of the effect of the intervention on treatment of cough and fast breathing with amoxicillin and diarrhoea with ORS/zinc at the drug shop were 2.8 (2.0–3.9), and 12.8 (4.2–38.6) respectively. From the household survey, the prevalence ratio of the intervention effect on use of RDTs was 3.2 (1.9–5.4); Artemisinin Combination Therapy for malaria was 0.74 (0.65–0.84), and ORS/zinc for diarrhoea was 2.3 (1.2–4.7).

**Conclusion:**

iCCM can be utilized to improve access and appropriateness of care for children at drug shops.

## Introduction

After the first month of life, about half of mortality in children under five years of age in sub-Saharan Africa is caused by malaria, pneumonia and diarrhoea. [1] These three illnesses usually manifest as an acute febrile illness, with overlapping symptoms. Following on from lessons learned in the public sector roll out of Integrated Management of Childhood Illness (IMCI) [2], [3], the integrated Community Case Management (iCCM) of malaria, pneumonia and diarrhoea targets the community level for integration of diagnostics with pre-packaged drugs for these illnesses. iCCM is now being scaled up through the efforts of UNICEF, WHO and several large donors. [4]

Under the iCCM strategy, lay community members are empowered to diagnose and treat malaria, pneumonia and diarrhoea using diagnostics (malaria rapid diagnostic tests - RDTs - and respiratory timers) and the dose specific pre-packaged drugs: artemisinin combination therapy (ACTs), dispersible amoxicillin tablets and oral rehydration salts/zinc sulphate (ORS/zinc) respectively. Consequently, Uganda [5] and other low income countries have now adopted a policy of iCCM implemented primarily through Community Health Workers (CHW).

However, about 60% of parents with febrile children in Uganda first seek care in the private sector, especially at drug shops. [6] Unfortunately, the standard of care in these drug shops is poor. [7], [8]

Recognizing the high utilization of the private sector and drugs shops for fever treatment in low income countries, the Global Fund through the Affordable Medicines Facility – Malaria (AMFm) in 2010 subsidized ACTs for both the public and private sector in a pilot study in 8 countries. [9] An independent evaluation of the AMFm pilot concluded that subsidies combined with supporting interventions were effective in rapidly improving availability, price and market share of quality assured ACTs in the private-for-profit sector. [10], [11] However, the lack of programmatic integration of RDTs in the AMFm presumably led to widespread presumptive treatment of fever with anti-malaria drugs only.

While the AMFm may have encouraged presumptive treatment of fever with antimalaria drugs, malaria RDTs are now available, allowing rapid detection of “RDT-negative fever”, prompting the need for alternative appropriate treatment. Also, WHO now recommends parasitological diagnosis of malaria for all patients, including children less than 5 years, prior to treatment. [12]The challenge now is to integrate both diagnostics, and alternative appropriate treatment, [13] in order to simultaneously achieve rational use of drugs for both antimalarials and antibiotics, at the same time as good quality of care for the febrile child, irrespective of cause of fever.

As drug shops remain a major source of care for febrile children in low income countries, there is a need to study interventions aimed at improving their quality of care in the management of common febrile childhood illnesses. We set out to assess the feasibility and effect on access and appropriateness of treatment when we introduce diagnostics (RDT and respiratory timers) and promote pre-packaged paediatric-dosage drugs for acute febrile illnesses (malaria and pneumonia) and diarrhoea at private sector drug shops.

## Methods

### Study site

The study was conducted in two rural neighbouring districts of eastern Uganda located approximately 150 km north-east of the capital Kampala: Kaliro district (est. 216,000 inhabitants 2010) as the intervention district and Kamuli district (est. 270,000 inhabitants 2010) as the non-intervention or comparison district. This is a high malaria transmission area with estimated parasite prevalence of over 60% in school-age children. [14] These districts were also chosen because they had both participated in a previous study, the Consortium for ACT Private Sector Subsidy (CAPSS) pilot study where ACTs for malaria had been made available in registered drug shops. [15]

### Study design

This was a quasi-experimental study in one intervention and one non-intervention or comparison district using a plausibility design with before and after measurement, in line with current WHO/Alliance for Health Policy and Systems research recommendations. [16] The chosen plausibility design comparing two entire districts was deemed the most suitable given that the registered drug shops are supervised from district level, and that both supervisors and patients would likely have contaminated the study if individual drug shops had been randomized in a probability design.

This was a two phased study. In the first phase, prior to the intervention, we set out to determine the community care seeking and treatment practices as well as drug-seller treatment practices. Given that appropriateness of treatment for children with fever, cough with fast breathing or diarrhoea within the community and at drug shops was not known, we set out to determine these.

In phase two, (one year later after the intervention) we then determined the effect of the intervention on access to care and appropriateness of treatment in the community as well as at drug shops.

### The intervention

There were three main components of the intervention: 1) provision of dose specific pre-packaged and subsidized drugs and diagnostics to registered drug shops drugs; 2) training of drug shop attendants and 3) a community awareness campaign.

Modelled on the public sector iCCM intervention for community health workers, all registered drug shops in the intervention district received the following: **subsidised drugs:** The drugs were pre-packaged as unit doses and included: dispersible ACTs from Uganda's AMFm pilot; amoxicillin tablets (dispersible); low osmolar oral rehydration solution (ORS) and zinc sulphate tablets (dispersible) which were sold at a mark-up of 50–80% (typically buying at USD 0.25 and selling at USD 0.38). **Free diagnostics:** malaria RDTs; respiratory timers and diagnostic algorithms/charts. **Training:** Two drug shop attendants per drug shop received a 5-day training on how to use the diagnostics and dispense pre-packaged drugs including daily clinical sessions in a public health facility. The training was conducted by the Ministry of Health using the national iCCM training manual for community health workers, adapted for drug shop attendants. Drug shop attendants were trained to perform RDTs in each fever case and count respiratory rate in each case of cough with fast/difficult breathing prior to dispensing the appropriate recommended treatment.

The non-intervention district continued the current practice of distributing subsidized ACTs only under AMFm support and guidelines, relying upon previous training. This previous training was conducted by Ministry of Health trainers and the (CAPSS) pilot study [15] which had ended just prior to the start of our intervention and focused on treatment of malaria. The non-intervention district had also received an awareness campaign on improved treatment seeking for febrile children which was conducted by the CAPSS pilot study. Additional awareness information on appropriate care-seeking was provided through the district/community radios in the non-intervention area during the intervention period.

The community awareness campaign in the intervention district was conducted by Population Services International (PSI)/Programme for Accessible Health Communication and Education (PACE) in Uganda. This included branding of the drug shops, communicating with caretakers of children and providing information at markets, public gatherings and on community radios, on appropriate care-seeking.

**Figure 1:**
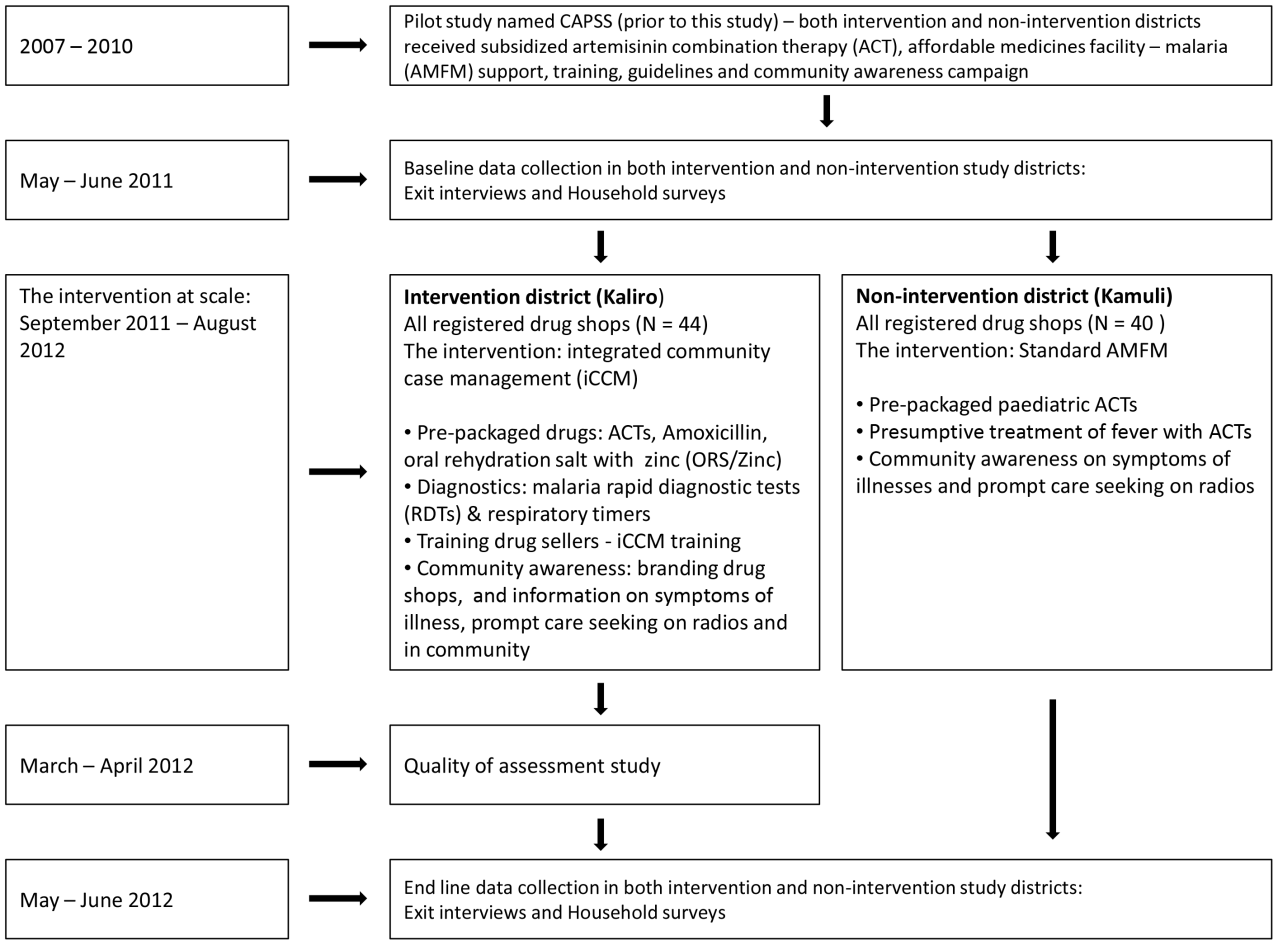
Study design and timing of interventions and data collection.

The intervention began in August 2011 and was implemented at full scale from September 2011 until August 2012. Fig. 1 shows the study design and timing of the intervention and data collection.

### Eligibility criteria

All 84 drug shops registered by the Uganda National Drug Authority (NDA) in the intervention (44 shops) and non-intervention district (40 shops) were included in the study. Un-registered drug shops, small shops and mobile medicine vendors were excluded as required by the NDA.

All caretakers and children for whom care was sought in a drug shop or who were residing in the participating districts were eligible to participate in the study.

### Data collection

Data collection was conducted both at baseline from May–June 2011 and at follow-up from May–June 2012 in both the intervention and non-intervention districts, using structured questionnaires. A 5-day training was conducted for data collectors including a pilot assessment outside the study clusters.

We conducted household interviews for community treatment practices (1604 before/2155 after) and exit interviews at drug shops for drug seller treatment practices (163 before/780 after).

#### Exit interviews at drug shops

All clients exiting the drug shops were approached and asked to participate in an interview using a semi-structured questionnaire, if they had come to the drug shop seeking treatment for a child less than 5 years of age. Data collectors were at the drug shop all day, from 8:30 am–7:00 pm for a total of four weeks. We also recorded information on the medicines purchased including the drug name, dosage and duration of treatment.

#### Household survey

A two stage cluster sampling using probability proportional to population size was used to select 1604 households in both the intervention and non-intervention districts at baseline and 2155 households at follow up, with children less than 5 years of age. At the first stage a probability sample of 30 villages/clusters were sampled, the same before and after. At the second stage, individual households were sampled from each cluster, not identical before and after. The study team randomly identified a starting point from a list of households obtained from the local leaders (village level household enumeration lists for local elections) and thereafter sampled every fifth household with children less than 5 years of age. The main caretaker (usually the mother) aged 15 years and above was interviewed face-to-face using a semi-structured questionnaire, designed to elicit treatment and care seeking practice for the most recent illness, less than 2 weeks prior to the interview. If there was no child under 5 years of age at the sampled house, the very next house was visited.

#### Direct observation

In order to directly assess quality of management of sick children by drug sellers, we randomly selected half of the participating drug shops in the intervention area and directly observed the drug sellers manage 2–3 sick children each. A field supervisor (a nurse) trained in iCCM, observed the drug sellers. This person recorded all the presenting complaints of the children observed, the assessment done by the drug seller and treatment given. At the same time, she made her own assessment of the child and recorded this.

### Sample size calculation

This was a two phased study. During phase one, sample size for the household survey was calculated to determine prevalence of appropriate treatment of sick children in the study area which was previously unknown and so was taken as 50%, using 95% confidence interval; a 5% margin of error and a design effect of 2. For exit interviews, data from all children with fever or cough with fast/difficult breathing or diarrhoea, for whom care was sought at all participating drug shops (both intervention and non-intervention combined) was collected, in order to estimate appropriateness of treatment provided at drug shops as previously published [7].

Phase 2: Informed by the baseline findings, the sample size (end line) for household survey was calculated based on baseline prevalence of appropriate treatment of fever and cough with rapid/difficult breathing, assuming 50% increase in correct treatment between baseline and end line, a 5% margin of error and a design effect of 2.

The outcome of interest was “appropriate treatment” of children in the community with each of the three symptoms: fever, cough and rapid/difficult breathing, or diarrhoea. Appropriate treatment was thus defined as: a child with fever in the community treated with ACTs; a child with cough and rapid/difficult breathing treated with amoxicillin and a child with diarrhoea treated with ORS/zinc.

For the exit interviews, end line sample size was calculated based on the prevalence of appropriate treatment of children at drug shops of 10%; 100% increase in appropriate treatment of children (i.e. 10% to 20%); 95% confidence level; and a 5% margin of error. For logistical reasons, in order to answer a different objective on community adherence to drugs, the sample size at end line in the intervention area is higher than that in the non-intervention area.

### Data analysis

The data was entered in Epi Data (www.epidata.dk), and was analysed using stata version 12 (www.stata.com). Baseline and follow up data was analysed separately and the baseline findings of the 2 districts combined have now been published. [7]

For this paper, survey-adjusted generalized linear models (log transformation and binomial distribution) and difference-in-difference analysis was used for both exit interviews and household surveys data. Descriptive statistics were generated separately for the intervention and non-intervention districts at each of the data collection rounds (before and after) and the differences between/within intervention and non-intervention areas are presented in terms of p-values. Survey-adjusted differences in management of children before and after the intervention were also calculated with intervention status as the exposure. Finally, survey-adjusted prevalence ratios of the intervention effect on appropriate treatment of malaria, pneumonia and diarrhoea were derived, adjusting for confounding. In the step-wise multivariate regression analysis, covariates were included in the model in descending order of their strength of association with the outcome variable in univariate analysis (p value < = 0.1). If inclusion of a covariate into the model produced a>10% difference in the prevalence ratios, it was considered a confounder and left in the model. In the final models, the presence of interaction was also assessed. For the difference-in-difference analysis, an interaction term for intervention status and time of assessment (baseline or end-line) was included in the models and if significant, the coefficient of the interaction was presented.

### Ethical approval

Ethical approval was obtained from Uganda National Council of Science and Technology (# HS 1184), the Uganda National Drug Authority (# 0456/ID/NDA) and Makerere University School of Public Health (# 166). Written consent was obtained from the child caretaker who was interviewed during the household survey and exit interviews.

We report according to the Strengthening the Reporting of Observational Studies in Epidemiology (STROBE) Statement. [17]

### Role of the funding sources

The Einhorn Family Foundation had no role in study design, data collection, data analysis, interpretation or writing the report. Medicines for Malaria Venture participated in all the above stages. The corresponding author had full access to all the data in the study and had the final responsibility for the decision to submit for publication.

## Results

We completed 3759 (1604 before/2155 after) households interviews and 943 (163 before/780 after) drug shop exit interviews with caretakers of children less than 5 years of age. From the baseline household survey, the comparison areas (districts) were generally similar before the intervention with some differences only in terms of the source of water used and the head of the household (Table 1).

**Table 1:** Characteristics of participating children and caretakers from household survey.

	BEFORE			AFTER	
	Non-Intervention	Intervention	p-value	Non-Intervention	Intervention
	N = 811	N = 794		N = 1076	N = 1079
	n (%)	n (%)		n (%)	n (%)
Child's gender – male	371 (48.2)	392 (49.9)	0.5	516 (48.9)	548 (51)
Caretaker's highest level of education - primary school	451 (55.7)	457 (57.6)	0.4	637 (59.2)	668 (61.9)
Marital status – married	744 (91.7)	725 (91.3)	0.8	952 (88.5)	992 (91.9)
Caretaker's employment – subsistence farmer/housewife	726 (89.5)	727 (91.6)	0.2	993 (92.3)	1029 (95.4)
Floor type – hard earth/mud	625 (77.1)	623 (79.6)	0.2	828 (77.1)	904 (83.8)
Main type of fuel for cooking – firewood	745 (91.9)	745 (93.8)	0.1	985 (91.5)	1,015 (94.1)
Main source of water – bore hole	691 (85.2)	737 (92.8)	<0.01	953 (88.6)	1,022 (94.7)
Head of household – partner/husband*	716 (88.4)	607 (76.5)	<0.01	927 (86.2)	869 (80.5)
**Continuous variables**	**Mean (SD)**	**Mean (SD)**		**Mean (SD)**	**Mean (SD)**
Child's age (months)	18.1 (13.6)	17.6 (13.1)	0.5	20.3 (14.2)	17.5 (12.2)
Caretaker's age (years)	29.5 (8.6)	30.1 (9.7)	0.2	28.5 (7.9)	27.9 (8.1)

*This reflects that the caregiver is often not the head of the household.

From drug shop exit interviews, all febrile children were treated presumptively with malaria drugs in both intervention and non-intervention areas before the intervention and there were no diagnostic tests used prior to treatment. At follow-up, while no child with fever received any diagnostic test in the non-intervention district drug shops, nearly 90% in the intervention district had a parasitological diagnosis of malaria with an RDT and over half with symptoms of cough and fast breathing first had their respiratory rate counted. Treatment of diarrhoea with the recommended drug ORS/zinc, in the intervention district drug shops was 77% compared to only 5% in the non-intervention district, Table 2.

**Table 2:** Symptoms and management of fever, cough with fast breathing and diarrhoea in children below 5 years of age at drug shop exit interviews (survey adjusted).

	Non-intervention		Intervention		Difference	
	Before	After	Before	After	% (95% CI)	
	N = 83	N = 283	N = 80	N = 497		
Management	n (%)	n (%)	n (%)	n (%)		p-value
**Children who had fever**	71/83 (85.5)	275/283 (97.2)	74/80 (92.5)	487/497 (98.0)		
- Malaria RDT used to make diagnosis	0/71 (0)	0/275 (0)	0/74 (0)	427/487 (87.7)	87.7 (79.0–96.4)	<0.0001
– Malaria RDT used and ACT dispensed	0/71 (0)	0/275 (0)	0/74 (0)	343/487 (70.4)	70.4 (60.4–80.4)	<0.0001
**Children with cough & fast breathing (pneumonia)**	8/83 (9.6)	45/283 (15.9)	24/80 (30)	73/497 (14.7)		
- Amoxicillin (5–7 days) dispensed	0/8 (0)	12/45 (26.7)	0/24 (0)	55/73 (75.3)	48.6 (44.3–53.1)	<0.0001
- Respiratory timer used	0/8 (0)	0/45 (0)	0/24 (0)	40/73 (54.8)	54.8 (33.8–75.8)	<0.0001
- Respiratory timer used and amoxicillin dispensed	0/8 (0)	0/45 (0)	0/24 (0)	36/73 (49.3)	49.3 (27.4–71.2)	<0.0001
**Children with diarrhoea**	25/83 (30.1)	111/283 (39.2)	31/80 (38.8)	176/497 (35.4)		
- ORS and zinc dispensed	0/25 (0)	6/111 (5.4)	0/31 (0)	136/176 (77.3)	71.9 (67.7–74.1)	<0.0001

**Figure 2:**
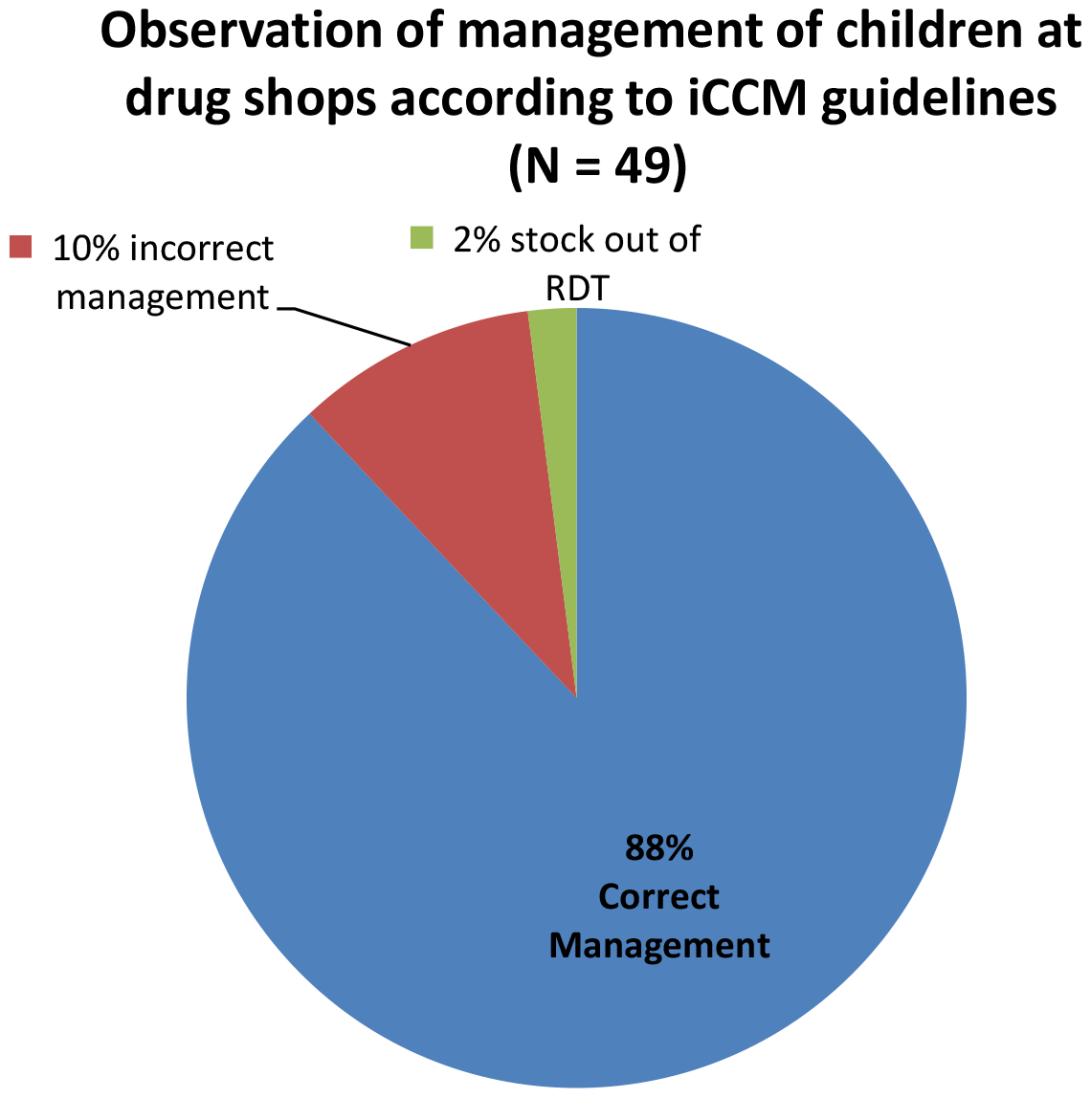
Quality of assessment of children at drug shops, from direct observations in the intervention area.

The effect of the intervention at the level of the drug shop was: almost total replacement of the obsolete antibiotic cotrimoxazole from the intervention area, three times better access to amoxicillin, the recommended drug for pneumonia and a reduction in overall antibiotic use by 18% in the intervention area as compared to the non-intervention area (Table 3). In addition, there was thirteen times better access to ORS/zinc for diarrhoea in the intervention district compare to the non-intervention district. From the direct observation of drug sellers, 88% of sick children presenting at drug shops with fever, cough or diarrhoea were appropriately managed according to the iCCM guidelines (Fig. 2; Table 4). Results similar to those obtained from the direct observation exercise were obtained during repeated routine support supervision visits.

**Table 3:** The effect of the intervention on treatment using antibiotics, ACTs and ORS/zinc, by survey-adjusted prevalence ratios using difference in difference analysis and generalized linear models.

	Non-intervention		Intervention		Prevalence ratios
	Before	After	Before	After	PR (95% CI)
	N = 83	N = 283	N = 80	N = 497	
Management	n (%)	n (%)	n (%)	n (%)	
Children with pneumonia treated with amoxicillin (5–7 days)	0/8 (0)	12/45 (26.7)	0/24 (0)	55/73 (75.3)	a2.8 (2.0–3.9)
Children with pneumonia treated with cotrimoxazole	3/8 (37.5)	11/45 (24.4)	19/24 (79.2)	2/73 (2.7)	0.07 (0.01–0.39)
Overall antibiotic use	54/80 (65.1)	208/283 (73.5)	36/86 (45.0)	298/497 (60.0)	0.82(0.69–0.97)
Children with fever treated with ACT	27/71 (38.0)	d113/275 (41.1)	12/74 (16.2)	393/487 (80.7)	c ^,^4.2 (1.9–9.4)
Children with diarrhoea treated with ORS/zinc	0/25 (0)	6/111 (5.4)	0/35 (0)	136/176 (77.3)	a ^,^ b12.8 (4.2–38.6)

Note: Adjustment for various possible confounders including age, caretaker's gender, caretaker's highest education, employment etc did not change most results. Adjusted results are reported below only where changes were statistically significant.

a Computed between intervention and control, at end-line only because of presence of zero cells at baseline.

b Adjusted for child-age (un-adjusted  = 14.3, 95% CI 4.8–42.4).

c Adjusted for employment status (unadjusted  = 4.1, 95%CI 1.8–9.2).

d Additional febrile children were treated with quinine (23%), chloroquine (5%) and sulfadoxine/pyrimethamine (4%).

**Table 4:** Appropriateness of management of children at drug shops, from direct observation in the intervention area (N = 49).

Management	n (%)
**Fever management**	
Complained of fever	47/49 (96)
Number who received malaria RDT test	44/47 (94)
Positive malaria RDT	33/44 (75)
Received recommended treatment with ACTs	33/33 (100)
Number with negative RDT who received anti-malaria drug	1/11 (9)
**Pneumonia management**	
Complained of cough	30/49 (61)
Respiratory rate counted	30/30 (100)
High respiratory rate (diagnosis pneumonia)	25/30 (83)
High respiratory rate and received amoxicillin	24/25 (96)
High respiratory rate and no amoxicillin	1/25 (4)
Low respiratory rate and no amoxicillin given	5/5 (100)
Challenge with counting respiratory rate	2/30 (7)
Respiratory rate counted when no cough	4/49 (8)
**Diarrhoea Management**	
Children with diarrhoea	14/49 (29)
Children with diarrhoea who received ORS/Zinc	13/14 (93)

The effect of the intervention at household survey level was: three times better access to malaria rapid diagnostic tests for children reported to have fever in the intervention district compared to the non-intervention district (Prevalence ratio 3.2, 95% CI 1.9–5.4) and about 30% less use of artemisinin combination therapy in the intervention district for fever in children as compared to the non-intervention district (Prevalence ratio 0.74, 95% CI 0.65–0.84), Table 5. There was also decreased use of the obsolete antibiotic cotrimoxazole for reported pneumonia symptoms in the intervention area compared to the non-intervention district (Prevalence ratio 0.45, 95% CI 0.27–0.47) while treatment of diarrhoea with ORS/zinc was two times better in the intervention district as compared to the non-intervention district (Table 5).

**Table 5:** Survey adjusted appropriateness of management of illness within the last 2 weeks, from household survey.

Management	Non-intervention		Intervention		
	Before	After	Before	After	Prevalence Ratio (CI)
	N = 457	N = 711	N = 483	N = 748	
	n (%)	n (%)	n (%)	n (%)	
**Children who had fever**	426/457 (93.2)	695/711 (97.7)	452/483 (93.4)	718/748 (96)	
- Malaria RDT used to make diagnosis	-	30/695 (4.3)	-	99/718 (13.8)	3.2 (1.9–5.4)
- Parasitological test for malaria performed (RDT or microscopy)	-	133/695 (19.1)	-	170/718 (23.7)	1.2 (1.01–1.5)
- Received Artemisinin combination therapy	203/426(47.7)	468/695 (67.3)	152/452 (33.6)	362/718 (50.4)	0.74 (0.65–0.84)
**Children with cough + fast breathing (pneumonia)**	130/457 (28.4)	216/711 (30.4)	143/483 (29.6)	219/748 (29.3)	
- Respiratory rate timer used	0	0	0	9/219 (4.1)	
- Cotrimoxazole treatment	120/184 (65.2)	155/278 (55.8)	84/193 (43.5)	46/219 (21.0)	0.45 (0.27–0.74)
- Amoxicillin treatment	40/130(30.8)	62/216 (28.7)	45/143 (31.5)	56/219 (25.6)	0.82 (0.58–1.2)
**Children with diarrhoea**	246/457 (53.8)	387/711 (54.4)	270/483 (55.9)	432/748 (55.1)	
- ORS and Zinc treatment	0	10/387 (2.6)	0	26/432 (6)	2.3 (1.2–4.7)

Treatment seeking at a government health facility for children with an illness less than 2 weeks prior to the interview remained the same between baseline and follow-up in the intervention and non-intervention districts (Table 6). In the intervention district, utilization of registered drug shops increased from 29.4% at baseline to 55.1% at follow-up and this increase came mainly from unregistered drug shops and other informal private sector.

**Table 6:** First source of care for children with an illness less than 2 weeks prior to interview, in the household interviews.

	Non-intervention			Intervention		
	Before	After	p-value	Before	After	P value
	N = 457	N = 711		N = 483	N = 748	
	n (%)	n (%)		n (%)	n (%)	
Government health facility	84 (18.3)	127 (17.9)	0.8	101 (20.9)	154 (20.6)	0.9
Managed at home	149 (32.6)	305 (42.9)	<0.001	79 (16.4)	130 (17.4)	0.7
Drug shop	137 (30)	246 (34.6)	0.1	142 (29.4)	412 (55.1)	<0.001
Other private sector*	72 (15.8)	31(4.4)	<0.001	145 (30.0)	41 (5.5)	<0.001
Others	15 (3.3)	2 (0.3)	<0.001	16 (3.3)	11 (1.5)	0.04

*his was mainly the informal/unregistered private sector.

## Discussion

We demonstrate that it is possible to adopt and utilize iCCM for management of fever in children in private-sector registered drug shops to improve access and appropriateness of treatment.

At follow up, nearly 90% of febrile children who sought care at registered drug shops in the intervention district received a malaria rapid diagnostic test and more than half of children with cough and fast breathing first had their respiratory rate counted prior to receiving treatment. About three quarters of children with diarrhoea received the recommended treatment with ORS/zinc.

Furthermore, from both household surveys and exit interviews, overall antibiotic use reduced in the intervention area and the use of the obsolete antibiotic, cotrimoxazole, for treatment of pneumonia was greatly reduced (97% at the level of drug shops and 55% at population level). Also, from the household survey, parasitological diagnosis of malaria was higher in the intervention area contributing partly to the lower use of ACTs there.

While we found increased utilization of drug shops in the intervention district, this increase came from people already utilizing the private sector with a shift mainly from unregistered drugs shops and vendors to the participating, registered drug shops. The overall utilization of government health facilities (rural health centre and hospital) as the first source of care remained the same in the intervention district both at baseline (20.9%) and at follow up (20.6%). We could therefore allay fears in this context that interventions at drug shops will distort the health system at the expense of the public sector. [18], [19] Similar results have been shown from Kenya after intervention with subsidized ACTs in retail shops. [20]

The proportion of children managed at home in the intervention district remained the same both at baseline (16.4%) and at follow up (17.4%) and increased in the non-intervention district. This indicates continued need for interventions targeting caretakers for better home care, e.g. by means of Community Health Workers and iCCM.

Recently, there has been considerable debate about the role of the AMFm in improving access to quality malaria treatment in low income countries through drug shops. [10], [15], [18], [21], [22], [23] The Global Fund board decided in November 2012 to integrate the AMFm into core grant management and financial processes. [22] From 2014, countries will now apply for funding to subsidize ACTs through their usual Global Fund applications. In many cases these efforts will be combined with malaria RDT introduction in private sector. As countries enter this next phase of private sector intervention, it will be imperative to ensure correct management of febrile children with appropriate differential diagnosis and treatment also of non-malaria fevers.

The iCCM strategy is effective in reducing both morbidity and mortality in febrile children. [24], [25], [26] However, there are numerous challenges with scaling-up iCCM, especially around the sustainability of the intervention in terms of salaries and motivation of community health workers and national ownership of the intervention [27]. In some countries like Uganda, only 30% of the districts have trained and active CHWs. Private sector drug shops will therefore remain an important source of care for the foreseeable future. Involving them in the campaign for better care for children is a potential game changer that could contribute to lowering child mortality. Private and public sector care must both be embraced and enhanced to ensure well-functioning pluralistic health systems. [28], [29], [30]

Care should be taken when interpreting and generalizing these results. First, we used one intervention and one non-intervention district in a high malaria transmission area in Uganda and collected data at two time points, at baseline and at follow up. However, the use of multiple methods of data collection - exit interviews, direct observation and household surveys - which produced similar results improves the strength of the results. Secondly, the malaria rapid diagnostic tests used in this study were provided free of charge so different subsidy levels for RDTs need to be explored for iCCM at drug shops. Finally, we did not cross check the diagnostic test results real time during the exit interviews in order not to interfere with drug seller practice. However, we conducted the quality of assessment exercise where a nurse trained in iCCM observed the drug sellers diagnosing and treating children and then re-assessed the children, for comparison. This quality of assessment exercise showed about 90% correct management of children by drug sellers.

This study shows that in high malaria prevalence areas in rural Uganda, the iCCM strategy may be adapted for utilization at registered drug shops to diminish the presumptive treatment of malaria and to increase the integrated assessment and care of children with fever. AMFm and other private sector initiatives should follow the path of the public sector from presumptive, vertical management of malaria only to integrated management of the sick child. Further research is necessary in low malaria prevalence areas and on methods for cost recovery of RDTs.
